# Suicide Exposure in a Polymediated Age

**DOI:** 10.3389/fpsyg.2021.694280

**Published:** 2021-07-27

**Authors:** Jo Bell, Christopher Westoby

**Affiliations:** ^1^Faculty of Health Sciences, University of Hull, Hull, United Kingdom; ^2^Faculty of Arts, Cultures and Education, University of Hull, Hull, United Kingdom

**Keywords:** suicide, exposure, social media, polymedia, prevention, new media, lived experience, qualitative

## Abstract

With growing evidence that media plays a vital role in shaping public understanding of suicidality and influencing behaviours, media portrayals of suicidality have for some time been the focus of suicide prevention efforts. Traditional media has changed, and now exists alongside and within an instantaneous, interactive sharing of information created and controlled by anyone; the way most people use it today incorporates a wide variety of online communication media. *Polymedia* describes media communication as both a product and process, where anyone can contribute and act as producers, consumers, audiences, and critics. In a “Polymediated age,” media exposure becomes much more complex. To understand how media exposure to suicide influences and impacts on others, it is important to take into account the *communicative ecology of media technologies* and the different interactions we can have with them. We researched the effects of this type of exposure by conducting in-depth interviews with a purposive sample of individuals who have lived experience and/or knowledge of suicide exposure via polymediated communication in the aftermath of a suicide. Using thematic analysis, our data demonstrates how exposure to suicide has become more complex as a result of new communicative media technology: it can be both a gift and a curse, difficult to distinguish, predict or control. Polymedia has the power to determine new forms of narrative and new forms of behaviour that on the one hand can provide support and prevention efforts, while on the other hand can promote conflict and cast an adverse influence on suicidal behaviour. Polymedia provides novel affordances for very intimate collective exposure to suicide. Our findings shed important new light on how the interplay between news media and social media has transformed our relationship with the information to which we’re exposed. We highlight important suggestions for those working in suicide prevention to develop (1) media strategies that recognise the multiple ways in which users are exposed and impacted, and (2) mechanisms for a strategic amplification and moderation of specific types of content. Media organisations and users of social media alike can contribute to maximising the beneficial capacity of polymediated exposure to suicide.

## Introduction

In the last 10 years social media has risen to immense popularity and come to play an important role in shaping norms and codes of practice globally. It is used for interpersonal communication and information seeking, providing users with information and allowing them to connect with people. In 2021, 4.2 billion people worldwide (53.6% of the global population) are active social media users (Data Reportal, 2021); the UK has 53 million active social media users (71.6% of the population) (Statista, 2021).

Social media, its influence and its embeddedness in our society is ever evolving. For example, for Generation Z, who were born after 2001, digital culture is a natural part of life; the internet is relied upon exclusively for everyday communication and entertainment, and has a more authoritative position than ever as the primary source of information (Turner, 2015; Pikhart and Klímová, 2020). We are currently witnessing a much wider shift, toward a more permanently connected digital culture, where “intelligent internet,” machine learning and artificial intelligence drive the connections we have with one another and the wider society, cultures and consumerism. Web 4.0, known as the symbiotic web, reflects this notion of a symbiotic interaction relationship where our devices are always on, we are always connected, and systems are learning to understand users via artificial intelligence and advanced, often opaque algorithms.

Alongside this cultural shift, literature on the topic of social media and its impact on various aspects of human life has highlighted both the negative and positive influences of intense social media use on individuals and society (Twenge, 2017; Pinker, 2018; Appel et al., 2020). In this paper, we draw attention to the benefits and risks associated with extensive media use in relation to suicidality, specifically to the context of the aftermath of a suicide.

Growing evidence suggests that exposure to suicide via media, including traditional, digital, and social media, can play a vital role in shaping public understanding of suicide, and can influence actual behaviours. It is now widely accepted that sensationalist forms of media coverage of suicide and suicidal behaviour can trigger further suicides (Niederkrotenthaler, 2017). Recent research has also suggested that audiences can also be impacted in positive ways when exposed to content on how to cope with suicidality and adverse circumstances (Niederkrotenthaler et al., 2010, 2020a). The effects of exposure via traditional media have received much research attention (e.g., Stack, 2003; Gould, 2006; Neiderkrotenthaler and Stack, 2017), along with several studies that have explored the impact of exposure via suicide-related internet use (Bell et al., 2017; Biddle et al., 2018), suicide-related educative websites (Till et al., 2017), online suicide message boards (Niederkrotenthaler et al., 2016) and online videos (Dagar and Falcone, 2020; Niederkrotenthaler et al., 2020b). However, much less is known about the impact of exposure via new online communication technologies such as social media. Research in this area to date is extremely limited.

We discussed the positive and negative impact of social media use (e.g., Facebook) in the aftermath of a suicide in our previous work which examined perspectives from lived experiences (Bailey et al., 2014; Bell et al., 2015; Bell and Bailey, 2017). On the positive side, our findings highlighted how it was a means for the bereaved to help make sense of the event and manage the distress and grief associated with the death. Participants used sites (Facebook being the most common) to reach out to users in comparable circumstances of incredible shock, trauma and sadness to provide much-needed support for one another that lasted through the immediate aftermath of the suicide, continuing for many months, sometimes years after. Negative effects included the potential for alternative contested narratives, increased distress, conflict, and suicide contagion (Bell and Bailey, 2017).

Findings from our recent research (Bell and Westoby, forthcoming) indicated that it is now common for people to find out about a suicide via social media within hours of the event and for people to spread the news instantly by sharing and re-posting; this is consistent with findings reported by Heffel et al. (2015) and Robertson et al. (2012). Some participants in Heffel et al.’s (2015) study who were close to the deceased reported feeling angry at others for constantly posting on the deceased’s Facebook page; other participants found these reminders distressing. It became evident that much of the information exchanged in this way was inaccurate. Robertson et al. (2012) argue that rapid widespread rumour, speculation and information about the death facilitated by these technologies were likely to increase the risk of suicide contagion amongst young people.

Our findings were consistent with Sumner et al. (2020) in suggesting that the increased connection made possible through social media platforms such as Facebook expands the range of impact from suicide exposure in multiple ways, promulgating detailed and intimate information and sensationalising how the death is seen. More needs to be known about the positive and negative effects of this phenomenon.

At the same time, research examining the lived experience of media exposure to suicide is also very limited. Miklin et al. (2019) and others (e.g., Hjelmeland and Knizek, 2010) argue that this is an important gap in our understanding of why suicide happens and how people are affected and impacted by exposure to suicide. Miklin et al. (2019) suggest individuals may develop their own understanding of the suicide through interpersonal conversations, public health campaigns, media and individual contemplation. In this activity, a very intimate and personal experience with suicide – such as exposure to the death of a loved one by suicide – is significant because it forces one to engage in the process of meaning-making. Work by Neimeyer et al. (2006, 2014) has shown that this process is more often collective, rather than individual, as people draw from a variety of sociocultural resources and each other as they try to make sense of the death.

Thus to understand how media exposure to a death by suicide influences and impacts on others, it is important to take into account the *communicative ecology of media technologies* and the many different interactions we can have with them. This understanding must be rooted in the lived experience of those who have been exposed.

### Polymedia

Polymedia refers to the abundance of media sources that are interrelated in everyday conditions (Madianou and Miller, 2012); it signifies the many different forms that media can take and the many different interactions we can have with them. Social media, for instance, is one element of this abundant and complex weave of interrelated media sources.

The term polymedia was first proposed by Madianou and Miller (2012, 2013) to describe the emerging environment of proliferating communication opportunities and to understand the consequences of digital media in the context of interpersonal communication. They suggested that polymedia, with its emphasis on the affordances of the communicative environment, radically transforms interpersonal communication at a distance. This carries implications for the ways in which interpersonal communication is enacted and experienced.

Kudaibrgenova (2019) discussed the role of polymedia in shaping diverse discourses, pointing out that social media often spreads information ahead of conventional media and is used to actively contribute to a myriad of messages and agendas. Polymediated communication is both a product and a process, where anyone can contribute and individuals can act as producers, consumers, audiences and critics. In the context of news and information, the opportunities provided by polymedia mean that there are increased ways for the narrative to evolve. Firstly, certain content and discourses reach wider audiences much more rapidly. Secondly, audiences are no longer passive consumers of media, rather they are active participants being simultaneously consumers and producers of content (Herbig and Herrmann, 2016). By interacting with news information rather than passively consuming it, individuals spread the story further by sharing it, and generate content by commenting on it.

We argue that in a polymediated age, exposure to suicide becomes much more complex. When the creation of (suicide) content changes and spreads via the opportunities provided by polymedia, the audience is exposed to much more than the story in isolation, as would be the case in traditional news media. Rather, reactions, opinions, other experiences, beliefs and judgements are posted and reposted by users in response to the story. By doing so, the story is perpetuated by the audience; it is fluid and continues to evolve beyond its original form.

Madianou and Miller (2012, 2013) argue that polymedia also emphasises how users exploit these affordances in order to manage their emotions and relationships in the recursive process of creation. They suggest that this negotiation (and the social or emotional consequences) often becomes the message itself. This in turn raises important questions about the power of the content and the power of those who frame the agenda and the message online.

Polymedia theory has been utilised in a variety of contexts – in the field of Communication Studies, for example – but not (in so far as our own knowledge to date) in the context of exposure to suicide. However, we suggest that it provides a fruitful framework because of its emphasis on communicative ecology and the relationship between the technological and the social, and because, as Madianou (2012, 2014) points out, in polymediated communication, the ways in which users navigate media will often closely reflect their emotional and social needs.

In this article we draw upon qualitative data from in-depth interviews with a purposive sample of individuals who have lived experience and/or knowledge of suicide exposure via polymediated communication in the aftermath of a suicide. This included (1) individuals who have been bereaved and affected by suicide and (2) individuals working in services supporting those who are bereaved and affected by suicide.

We are concerned with how polymedia has changed the nature of exposure and the impact of the exposure on its audience. This presents several key questions. First: how does the news of a suicide start and spread? Second: how does the ensuing communication between users online perpetuate the spread of information? Third: how are the people who are exposed to this mass dialogue impacted? And finally: in what ways do new forms of communicative media differ from traditional media exposure?

Our analysis demonstrates how exposure to suicide has become more complex as a result of new communicative media technology (polymedia). In doing so we argue that exposure to suicide via social media can be a double edged sword (a gift or a curse). We illustrate the power of polymedia in determining new forms of narrative and new forms of behaviour that on the one hand can be leveraged to provide much needed support, disseminate reliable helping resources, and augment prevention and intervention efforts, while on the other hand can promote conflict and resentment and complicate the meaning-making process, which may cast an adverse influence on suicidal behaviour.

## Materials and Methods

The aim of the study was to gain a more detailed insight into the impact of polymediated exposure to suicide by exploring the various uses and practices of online social media communication in the aftermath of a suicide.

A panel of experts in supporting those affected or bereaved by suicide in some capacity and/or with lived experience of being bereaved or affected by suicide (i.e., those who were close to someone who died by suicide) was convened in February 2020. The panel attended a Knowledge Exchange workshop which was delivered by the authors, in collaboration with a regional Suicide Prevention Board (SPB) in the UK. The SPB consists of a range of experts responsible for the development and implementation of suicide prevention strategy in the region. An invitation to attend the workshop was circulated widely amongst contacts known to the SPB, appealing to those who had knowledge, experience and interest in the topic of social media use in the aftermath of a suicide.

The aim of the workshop was to bring together a purposive sample of experts from different professional backgrounds and with a wide range of experiences to discuss, reflect and share knowledge on the safe and responsible use of social media in the aftermath of a suicide. Amongst those that attended the workshop were a postvention service manager, police emergency responder, university student support service manager, regional suicide prevention lead, bereavement support worker, youth counsellor, school pastoral care manager, volunteer support worker, and experts in public and mental health (see Table 1).

**TABLE 1:** Workshop attendees by professional expertise and gender.

Gender	Profession/expertise
Male	Schools pastoral care manager
Male	Local authority schools safeguarding
Female	Local authority public health lead
Female	Police emergency responder
Female	Postvention support worker and service co-ordinator
Female	Local authority public health manager
Female	Postvention support service manager
Female	Mental health and well-being service team supervisor
Female	Children’s advocacy officer and lived experience
Female	Post graduate researcher/nurse
Female	Head of university student support service
Female	Youth counsellor
Male	Public health improvement officer

The workshop lasted for half a day. Topics discussed included the uniqueness of suicide bereavement; harmful and protective effects of social media use in the aftermath of a suicide; how social media can be harnessed to manage trauma, alleviate grief, and reach those who need support; how to mitigate against harmful effects and promote positive effects of social media use following a suicide. Discussions were guided by the facilitators (JB and CW), assisted also by the Suicide Prevention Lead and volunteer support worker with lived experience of suicide bereavement who were both members of the SPB.

All attendees completed a short qualitative evaluation survey at the end of the workshop. Using brief open-ended questions, the survey sought to establish how useful the workshop was perceived by experts to be in improving their understanding of the topic and how it might improve practice in their professional roles. At the end of the survey attendees were asked to indicate their agreement to take part in a follow-up in-depth individual interview with researchers. Attendees were also invited to nominate other known individuals with relevant lived experience (bereaved or affected by suicide) to take part in the interviews (facilitating a “snowballing” method of data collection for the researchers).

Notes were taken by the facilitators during discussions in the workshop and used as a basis to develop themes and questions for the interview guide. For example, if an issue or theme was raised and agreed upon by a significant proportion of experts at the workshop, regardless of professional background, then this would be noted as criteria worthy of further careful exploration at interviews. The workshops thus primed interview participants with respect to reflecting further on the impact of exposure to suicide via social media and their own personal and professional experiences ahead of the in-depth interviews.

The interviews (conducted online/telephone) sought to examine participants’ knowledge and experiences of polymediated exposure to suicide (including social media and how, when, and why this is used) and their perceptions of the impact of this on others in the community in much more detail. Semi-structured interview guides were used to illicit detailed information, with open-ended questions allowing for variations in the ways in which participants chose to construct and tell their stories. Ethical approval for the study was granted by the Faculty of Health Sciences Research Ethics Committee at the University of Hull, United Kingdom. Written consent was sought from interview participants prior to data collection. Regardless of their own professional background and experience, all participants were also provided with information regarding local and national accessible sources of support. All participants consented to interviews being recorded, and for their anonymised data to be used in publications. We have used pseudonyms here to protect individual identities.

All recorded interviews were transcribed verbatim by a professional transcribing service. Transcribed data were analysed by the authors using the constant comparative method, an analytical process associated with grounded theory (Glaser and Strauss, 1967) and thematic analysis (Braun and Clarke, 2006). Researchers were already familiar and sensitised to the data, having undertaken the interviews and facilitated discussions at the workshop. We began by coding the data within each transcript according to emergent themes using the principles of constant and continuous comparison of data. Each code was compared to previous codes to determine if a new code or theme was required or needed to be revised. This process was repeated reiteratively until themes were fully developed. Themes were constructed and decided upon the basis of consensus among all or most interviewees. In the following sections, we present these themes, which were common to most or all participants, using data from individual participants as illustrative examples along with written narratives constructed around them.

## Results

Table 2 shows anonymised details of interview participants. Eight interviews, lasting between lasting between 43 and 150 min in duration were undertaken. This yielded a data set of 589 min (almost 10 h) which constituted a total of 224 pages of qualitative text. In this paper we draw on data collected at the individual interviews and not data collected at the evaluation survey.

**TABLE 2:** Interviewees by gender, expertise and interview duration.

Gender	Expertise	Interview duration
Female (Cathy)	Professional and lived experience	150 min
Female (Lynn)	Lived experience	96 min
Female (Belinda)	Lived experience	68 min
Female (Anna)	Professional	66 min
Female (Isobel)	Professional	70 min
Male (Paul)	Professional and lived experience	50 min
Male (Isaac)	Professional	46 min
Female (Eve)	Professional and lived experience	43 min

### How the News of a Suicide Starts and Spreads

*Twenty years ago, your point of view was only in your little circle of friends, whereas now you can spread that across the world can’t you?* (Isaac 872)

All our interview participants reflected on the exposure and exploitation of suicide deaths on social media; Facebook in particular is the platform in which the majority of this activity occurred, but Snapchat, Instagram, and Twitter, including their instant messaging features, also contributed to the same overarching stories. When a death by suicide occurs, these platforms facilitate the swift spread of news across the country and beyond, frequently outpacing how quickly those close to the deceased can notify one another by traditional means (phone call, face-to-face conversation). Sharing is fast and easy; it can be done by anyone, anywhere. As one of our respondents noted, “The ripples go very far and wide” (Isobel 699).

How the news was first shared is not what most of our interviewees reported – this is often impossible to trace back – rather, it was how the news suddenly exploded. A like, comment or share of a post by one Facebook user will cause the post to appear on the newsfeed of their friends, friends of friends, and beyond. Any participation causes the reach of the narrative to widen much further. A Facebook user can tag their friend in a comment below the posted article, inviting them to join the discussion. With barely a tap or click, the sharing becomes exponential. A number of our interviewees recalled traditional media being the first to expose the suicide death to the spheres of social media. Others recalled traditional media being alerted to a suicide that is being spoken about by a relatively small community on social media, who then write an article about it, often lifting information by observing the community dialogue about it (at times even taking photos from the bereaved or deceased’s Facebook pages); this is both published online and then shared across social media platforms. The sharing of their articles to social media sites attracts (or provokes) comments from other users, which hands the control of the narrative from the old media to the consumer, ensuring its continued promulgation. Comments from social media users, whether divisive, offensive, or supportive, invite further participation from other users.

Isaac commented on the overwhelming number of different thoughts and opinions on a single story one is exposed to:

*Those comments that you click on then have clicks on them as well, so you’re suddenly*… *in an arena that you probably didn’t think you were going to be in*… *you’re then in a whole whirlwind of ideas and*… *thoughts.* (Isaac 469–872)

Isobel noted that some users would write a new post which lamented the passing of the deceased, or repost related content, and within this post they would tag said deceased, perpetuating the spread of the news:

*Some people’s accounts were very actively used and they would continue, as in they, family members, would continue to write messages and post, repost things constantly*… *the thing of tagging the person was common.* (Isobel 866)

As more people continue to contribute by sharing, posting, commenting, tagging and liking content related to the death, more are exposed. What would traditionally be contained within a relatively local network or community surrounding the deceased now reaches an (inter)national audience. A wide variety of people are connected by the news, from those who are closely related to the deceased to those who are distantly acquainted and everyone in between.

*What comes through your newsfeed is coming from lots of different directions, a lot of different age groups, a lot of different mix*…. *the easiest thing to do is share a meme, share a quote, share something quickly*…. *that’s how Instagram works, quick.* (Lynn 1367)

Lynn’s comment above illustrates what Eve refers to as the “general melee”: information coming from different directions, and numerous social media platforms all contributing to the story.

Anna, a bereavement support worker who responded in a school setting following the death of a pupil, reflects on the variety of channels being used by young people to communicate about the death (Snapchat, Facebook). The choice of platforms available to social media users doubles and triples the buzz of information bombarding the closely bereaved and distantly acquainted alike with condolences, fond memories, tributes, arguments, family and friendship group politics, distress, trauma, shock, disbelief, judgements, rumour, and speculation; simultaneously, more users are continually being alerted and exposed to the death.

*They all experienced everything in such different ways, they’d all heard so many different stories, different ways of him dying. There were some bits that didn’t quite add up, clearly. They all had different opinions on how things happened. And why things happened. Everyday different rumours and different stories.* (Anna 381)

A number of our respondents discuss how the spread of information is controlled, and to what extent. We refer to this as “control over the narrative” (Bell and Westoby, forthcoming). Paul demonstrated an acceptance of news spreading via social media before anyone can stop it.

*I contact schools and where schools have already known about it*… *they’ve found out on social media before they’ve found out from the local authority*… *It’s so widely acknowledged now that actually so much goes on social media first*… *we almost expect it.* (Paul 444–449)

The struggle for control over the narrative leaves some wondering whether it is their responsibility or right to intervene. Paul observed parents not wanting details of a suicide to be exposed on social media in view of their children; they felt that privacy was appropriate at such a time, and at such an age. However, the deceased student’s friends wanted to memorialise the death on social media. The school questioned whether it was their place to write a statement and whether to disable comments.

Paul is aware of the *status quo*; users of Facebook, including institutions, know that the spread of news via social media is unstoppable. It comes as a surprise but is at the same time predictable. The public have the power to perpetuate the story but are also powerless to stop it; it is a paradox of being both empowered and powerless.

### How the Ensuing Communication Between Users Perpetuates the Spread of Information

Our data suggests that in the wake of a suicide death, a perceived hierarchy is constructed by those affected. Many of our participants referred to what appeared to resemble a hierarchy of who has the right to grieve, who has the right to share information and expose others to it, who has the right to control the narrative, and who has the right to access and generate both the information itself and the manner in which it is used online. There is an inner circle of those who are very close to the deceased (usually their partner, their immediate family, and close friends), a wider circle of those who know the deceased quite well, and then a periphery of those who are more distantly acquainted or do not know the deceased.

Cerel et al. (2014) propose a nested model of suicide survivorship in the context of suicide bereavement. Our data points to a similar model of concentric circles radiating outward from the people closest to the incident, who are situated in the centre. Numerous references to “hierarchy,” “inner,” and “outer circles” by our respondents would suggest that social media users perceive a similarly shaped hierarchy in their online community.

One important facet of a nested hierarchy is the order in which people are informed of the death. With eager sharing comes the risk of users who are close to the deceased learning of the death for the first time via social media in a manner which lacks the sensitivity of hearing from a close family member or friend. Belinda recalls giving the news of her partner’s death via phone in order of who is in the inner circle: “You’ve got your initial layer of people you need to tell and then you’re working through it… I haven’t got to that next layer” (131). It is an example of an invisible yet perceptible hierarchical line being crossed: those closest to Belinda should be informed by phone, not social media (872). Not only is finding out from family preferred (offering a full conversation, a softened blow and reciprocated grief), it also confirms in the eyes of other users that you were close to the deceased. To learn via social media implies that your relationship with the deceased wasn’t significant. Paul expresses disappointment that he learned “through some friends that… passed the information on via Facebook” (52). Learning through social media about the death of someone one felt close to demotes them from the inner circle; they find out at the same time as everyone else in the outer circle.

Motives for each user’s participation in this spread varies from a well-intentioned tribute or raising of awareness to a vying for one’s own popularity and relevancy. One of our respondents used the term “bandwagoners” to describe people in the outer circles who capitalise on the news of a suicide to further their own profile on social media. A bandwagoner will make a display of their grief and publicly reflect on their connection to the deceased in attempt to be relevant to the situation, to draw attention to themselves and to harvest likes. This phenomenon recurred throughout our interviews:

*It’s the people who are just there for the moment, who just want to be a part of it. It’s the case of “oh I knew him, therefore I’m going to share it. It was like sharing and sharing and sharing and sharing and all for the likes.* (Lynn 161)

Lynn received personal messages from those trying to imply that they have a relationship with the bereaved as well as the deceased: “There was a lot of people like that… jumping on the bandwagon and messaging me all of a sudden” (127).

Isaac observed that people with only a vague connection to the deceased who strive for relevancy are looking for “their 5 min of fame” (687), which Isobel echoed:

*Even if they were very distantly friends with that person, [they] would*… *feel the need to kind of comment publicly to say they had some sort of link*… *that level of competitiveness*… *it’s almost like their fifteen minutes of fame*. (Isobel 231)

Isobel interprets the stretch to seem relevant to the deceased as comparable to how people talk about meeting a celebrity and using whatever method they could to identify themselves publicly as having some relationship to that person:

*So they would use that kind of*… *affiliation of “well*… *I met them and I knew them” and that may have been justifying their right to grieve but it might also have been that they sense that this person was this great person who had… ended their life and they wanted to be part of all that hype.* (Isobel 1041)

Eve observed that those involved in the scramble to be seen were “being crushed if they’re not at the top of the list” (Eve 166). This “list” refers to the hierarchy; she refers to the “quite abusive,” “bitter,” and “personal” nature of the exchanges. Paul refers to this as “that grief competition” (593), which Isobel confirms. Isaac (334) sees the inner circle as being impenetrable, evaluating one’s worth in terms of what knowledge they were privy to *before* the person took their life. “Somebody might post up a comment and then people would sort of compete for how well they know that person” (Isobel 266). Everyone lays their cards out (I know *this*. Well, I know *this*), who knew what, and for how long, “who was closest” and therefore “who’s got more right to be upset” (Isobel 53). These representations were sometimes disputed by others. Lynn described “bandwagoners” in the outer circle who sent messages recounting memories of her sibling who died by suicide as “extremely fake”: “I’m going to pretend I’m grieving, I’m going to pretend I’m hurting” (Lynn 246).

The outpouring of disinhibited emotion or opinion results in different and sometimes contested representations of those involved in the event and their actions. Conflicts arise from users whose depictions are coloured by their individual beliefs: people and their actions are condoned, condemned and analysed.

*Everybody has to react in their own way*… *they were angry and directing stuff*… *wanting to put things that are not correct on there*… *conspiracy theories and*… *who to blame.* (Belinda 270)

Cathy commented on people in the local community sphere and beyond who did not know her recently deceased son; they would express their opinions without considering that close family members can view this.

*Yeah, they’ve got no control over, over themselves*… *they don’t give a damn*… *They’ve just got to have their opinion*… *[They] don’t know the effect it can have.* (Cathy 2148)

### How Those Exposed to This Mass Dialogue Are Impacted

*It’s very kind of hit and miss I think isn’t it, with social media and how it helps, and how it doesn’t help?* (Anna 844)

The mass dialogue of polymediated communication forms a narrative made of many voices which we refer to as collective story-making. Exposure to collective story-making impacts users of social media in the different circles they exist in. People who are exposed are impacted in different ways. As Anna notes above, this can be a gift (offering consolation, help and support, which enables coping, meaning-making and shared grieving) and a curse (a toxic environment of drama that can lead to trauma, anger, distress, excluding and disenfranchising those in the peripheries, complicating grief and potentially contributing to suicide contagion). We begin this section by focusing on the negative impact which was underscored by many of our participants.

Firstly, our interviewees at times observed a lack of consideration for close family members from both traditional media and the online community alike. The news media sites publish an article detailed to an extent of the suicide being “sensationalised.” The publication of this inspires opinionated comments from the online community, the effect of which causes the family to lose their sense of control.

*That’s quite devastating, knowing that you and your family’s business would be splashed all over the local news. And then anybody and everybody could comment on that*. (Isobel 361)

Anna offers an example of when the representation of the actions of the deceased were contested, where “thousands and thousands of comments and different opinions on what had happened” collided (422). The impact of this contested representation “causes that conflict” and “spirals outward because of the intense emotions that they feel at that time” (908). She observed that the cumulative effect of exposure to the noise and melee was overwhelming:

*One young person sat up until three am in the morning scrolling through those comments, not sleeping, not eating because of what he’d seen on there*… *It definitely added that trauma for them to imagine what had happened, lots of pupils had dreams about what had happened*. (Anna 443)

Similarly, Isobel recounted working with close family members, who “found it very distressing to look at Facebook or to look at photographs of the person or to hear the person’s voice” (Isobel 613). As a result of this exposure, “they become so phobic that if they looked at it… they would go to pieces” (618).

Lynn reflects on how the behaviour of “extremely fake” bandwagoners impacted her. She described it as “triggering,” referring specifically to how it contributed to her own suicidality (“I was going to take my own life” [811]).

*Social media and everything going on with it adding and punching, it felt like punches*… *like drinking poison, more and more and more poison, it definitely contributed to my breakdown, and if that didn’t exist and I was a*… *lot more protected, well I certainly wouldn’t have had as much venom in me.* (Lynn 819)

Someone known distantly to Lynn used the story of her deceased sibling on their social media to further their status as a popular mental health blogger, an experience Lynn described as “traumatic.”

*She became Instagram famous with a mental health blog on Instagram*… *she was constantly talking about suicide*… *she had such a grand following*… *it became quite traumatic*… *it just felt like so much exposure over time*… *I had to block and delete her.* (Lynn 626)

Isobel observed different sides of the family (within the inner circles also) taking offence at the other’s posts: “I can’t believe that they’ve written on the wall because their relationship wasn’t like that” (284). There is perhaps an implied element of prestige to the public “wall” of the deceased, and people who write on it without (inner circle) authority defile it and cause upset to the inner circle. As Lynn said, “know your place”:

*People that had nothing to do with our family, nothing to do with [the deceased], nothing to do with me*… *or barely even knew him, having something to say*… *we don’t need you to talk*. (Lynn 385)

Eve offered the perspective of someone who is in the outer circle being exposed to content. Her own reaction to the death was unexpected: “it devastated me… completely blew me out of the water… I just couldn’t control myself” (243). She found herself compelled to visit the site but struggled to articulate why: “I was completely voyeuristic… I don’t know what made me keep being drawn to it… just [a] way of working through something I think for me” (Eve 315). Eve perhaps represents somebody who draws comfort from observing the events as they unfold but chooses not to contribute to the discussion.

Paul was “desperate” to know more about a recently deceased friend who he had lost contact with as a way of seeking closure (615). However, he didn’t feel as though he had a right to comment and so silently followed the conversations on Facebook.

Paul and Eve are part of a silent community of disenfranchised observers – people whose thoughts and feelings are not expressed, recognised or validated, perhaps due to a fear of judgement from others in the melee, a fear of causing further upset, or because “They didn’t know how to deal with the situation” (Lynn 578). In our data, we found instances of close friends being the quiet ones, suggesting that those in the inner circle are not immune to this reticence to speak. Those in the inner circle have the added pressure of being quite obliged *to* say something. The silence of this community makes it impossible to gauge their numbers.

All of our participants were able to identify ways in which polymediated exposure had positively impacted on the mental health of users; this included bringing people together to seek and provide emotional support, sharing information, exchanging happy memories and feeling comforted. For some it was a safe and accessible space for users to express their thoughts and emotions and gain acceptance and support from others, including signposting to helpful services and raising awareness. As Cathy notes, in essence, the positive impact came from the kindness that users shared: “There’s an awful lot of kindness out there” (2173). In the final part of this section, we shift our focus to this impact.

Paul refers to the feeling of warmth and how the family of his deceased friend found this platform “really helpful” in managing their emotions (70). Social media was seen as a place for memorialising; it seems to capture something positive about the deceased and their life. Paul illustrates an example of collective story-making that was cooperative, not full of contestations: “lots and lots of really positive and really lovely, lovely posts” (62) gravitated around Twitter and Facebook.

*It prompts people to share their memories within the comments, and I’ve done that also*… *All the normal, everyday stuff*… *insights into his life*… *comments from other people that you might not know*… *builds up a story of that person’s life.* (Paul 62)

Paul found that the 150 people building a positive legacy for his friend, and eulogising together, prevented him from feeling the self-blame and regret often inherent in the aftermath of a suicide. He found support, rather than distress, in the repeated exposure to the content, and was able to use the platform to engage in the process of meaning-making.

Similarly, Belinda fondly reflected on users who shared “positive quotes” and “affirmations,” while appraising the importance of their positive impact: these “are quite inspirational and have made me smile.” She asserted that this is “what I needed” (Belinda 751). Belinda recognised “the things that refer to people’s mental health” and in turn promoted this to others: “I share them on if I think they will help” (684). It is an example of the dissemination of helpful resources through users sharing and resharing.

Cathy put a post on her son’s Facebook page asking people to post memories, stories and photographs: “they’re lovely to me because they’re new… I’m never going to have a new photograph of [him]” (1346). She praised the support she received through Facebook, a support that was mutual between users, who would pay attention and reach out to one another when they recognised the “signs” (2636) that indicated distress.

For Eve, it felt as though the real world was failing her, so she turned to Facebook for solace:

*It devastated me*… *I remember going to the pub and not being able to stop crying for the night. And not knowing what way to take that. [So] it’s good to go on the Facebook page. And to see. People are still remembering him and what other people are saying, that was helpful. It was really warming to go on that and hear stories about him.* (Eve 232)

Anna explained how the school pupils later themselves attempted to take control of the narrative by setting up a private Facebook group which she described as a “coping mechanism” to the benefit of their emotional wellbeing:

*People could*… *post pictures, memories, video, all of that kind of thing*… *people would comment and say how they’re feeling on that day and what they remembered about the deceased as well*. (Anna 138)

Polymedia facilitated mutual support – a kind of collective meaning-making amongst the pupils, enabling them to “pull together,” express themselves, understand their emotions and the emotions of others.

*[Pupils shared an] understanding of they’re going through the same thing*… *I think it worked for them in knowing that they wasn’t alone*… *a lot of questions of well, why am I the only one that feels like this? And the answer was actually*… *you may experience emotions differently but you’re going through the same experience.* (Anna 762)

## Discussion

We have attempted to capture the communicative ecology of exposure by examining the ways in which the flow of content (what people are saying, when, where) changes the content itself. Traditional media was only one platform, one story, the monomyth (Herbig and Herrmann, 2016). Pre-social media, audiences would have been exposed to the news/details of a suicide in a less immediate and visual manner. Our analysis has highlighted how polymedia has changed the nature of media exposure to suicide. Exposure via polymedia does not begin and end with the publication of the story. It is dynamic rather than passive: the story continues to perpetuate and evolves beyond a linear trajectory as consumers share and comment on it, with different versions of events branching off in different directions, then branching further and so on. In this environment the audience is exposed to so much more. With polymedia, information spreads faster and there is a home for all of this information, imagery and intense emotion to gather in a place that is visible to everyone. The story is unfiltered and much more difficult to ignore or escape from. Rather than a monomyth, the consumers of the story are also the creators of a continuing narrative: “The process of invention occurs at both ends” (Herbig and Herrmann, 2016: 751).

Both the shared content and public comments which follow the story of a suicide death can influence how suicidality and suicidal behaviours are perceived, transmitted and responded to. As Pirkis et al. (2017) have argued, content may be interpreted very differently by different users, depending on a range of factors including their current mood: something they skip over at one point may be extremely salient for them at another. This complexity is amplified by the fact that online written text has low levels of social cues which can often lead to misunderstandings (Herbig and Herrmann, 2016). If content is highly emotive (as Madianou, 2012 and Madianou, 2014 suggests is often the case with polymediated communication in situations of unwanted exposure or scandal), there is more chance that elements of what is said will be misunderstood by the audience.

Polymediated exposure to suicide can be both a gift and a curse. For some the experience infers heightened vulnerability to suicide, for others it can help make sense of the death and infers a lesser likelihood of contagion. As Miklin et al. (2019) suggests, for some, witnessing the grief of others after exposure to suicide may generate a stronger commitment to life, making them more committed to not dying by suicide (the “I could never do it” group, p24) and more likely to seek help and appropriate support. How an individual will be influenced depends largely on the meaning they draw from the experience, which is now a collective, multifaceted, and rapidly shifting process. It does not lend itself to the simplistic casting in binary positive or negative terms; rather, it may be a complex combination which is difficult to distinguish, predict and control.

Despite this, there are some important and useful implications that we can infer. We have discussed the potential benefits of media discourse on suicidality highlighted by the Papageno effect in our previous work: we make recommendations for postvention services and users alike to flood social media with “cookie cut” statements that ask users to demonstrate respect and empathy and to pause and consider how their comments might impact on others before posting; such statements could be accompanied by stories of hope and resilience in the aftermath of a suicide (Bell and Westoby, forthcoming). Furthermore, we suggest that services could also share posts that link people directly to further resources and information about suicide prevention (Bell and Westoby, forthcoming).

It has been argued elsewhere that by maximising reporting and content on how to cope with suicidality and adverse circumstances, media can make a very relevant contribution to suicide prevention (Niederkrotenthaler, 2017 and Niederkrotenthaler et al., 2020a). There are some signs that once this intervention has been well established, applied AI and machine learning may offer some hope of carrying it on. Since 2017 Facebook has used machine learning and artificial intelligence to detect and flag posts – or comments on posts – that signal a high risk of suicide. Their machine learning is getting rapidly better at determining the suicidal intent of a post, using DeepText and multiple classifiers like time of day, type of content, material in comments, etc. When the concern threshold is hit, the next time the person who posted the concerning content logs in, they’ll receive links to help and resources and prompts to contact friends (Bell and Kasket, 2021). This is an operationalisation of the “Papageno effect.” It represents a form of what Donovan and Boyd (2021) refer to as “Strategic Amplification,” where Papageno-related content is detected, spread and amplified by algorithms, dampening the negative impact and remedying the spread of harmful content.

Our findings have shed important new light on how the complicated interplay between news media and social media has transformed our relationship with the information to which we are exposed, about which little is currently understood. However, being based on a small sample of individuals from an area in the north of England, our findings may have limited generalisability at present. Not all of our participants had direct lived experience, but rather worked closely with individuals who were directly affected. This sample was purposefully chosen to gain multiple perspectives, but its potential limitations should also be acknowledged. Further research of this type is needed to validate our analytical constructs and test the generalisability of our results. We need to extend our research to include representation from all participant groups; national, international and cross-cultural samples would provide increased depth and detail about how communication starts and spreads and strengthen our understanding of the impact of polymediated exposure. Future studies must include more individuals who have lived understanding, including those who work in media industries, professionals and practitioners who work in postvention services, and the wider circles of friends, acquaintances and observers.

Technology and the ways we interact with it shifts continuously. For this reason, it is also important that we continue to keep up with shifts and continue to gather new data on the topic, including data on how those exposed are impacted. It is essential that the views of ICT and media industry researchers are taken into account in future studies. Research would benefit from cross disciplinary initiatives that embrace fields such as artificial intelligence, machine learning, psychology, computer science and communication studies to elucidate new forms of knowledge.

Practices need to be updated to meet the challenges of networked media. ICT researchers must work with suicide prevention experts and media organisations to create and review policies, providing guidelines on what they should and should not amplify, and explore mechanisms for amplification.

Suicide prevention is everybody’s business: the public audience and those working in suicide prevention can each be part of the strategic amplifying of Papageno-related content. It is the responsibility of all who are involved in the flow of information. Education about responsible sharing of suicide-related content, proactive protective monitoring mechanisms for families, and closer collaboration between those working in media organisations and suicide prevention will help to maximise the beneficial capacity of polymediated exposure to suicide.

## Data Availability Statement

The datasets presented in this article are not readily available because all individual participants have been promised confidentiality. Requests to access the datasets should be directed to JB, j.bell@hull.ac.uk.

## Ethics Statement

The studies involving human participants were reviewed and approved by the Ethics Committee Faculty of Health Sciences at the University of Hull. The patients/participants provided their written informed consent to participate in this study.

## Author Contributions

JB conducted interviews. JB and CW undertook the data analysis, and conceived and wrote the manuscript. Both authors contributed to the article and approved the submitted version.

## Conflict of Interest

The authors declare that the research was conducted in the absence of any commercial or financial relationships that could be construed as a potential conflict of interest.

## Publisher’s Note

All claims expressed in this article are solely those of the authors and do not necessarily represent those of their affiliated organizations, or those of the publisher, the editors and the reviewers. Any product that may be evaluated in this article, or claim that may be made by its manufacturer, is not guaranteed or endorsed by the publisher.
